# Modelling the effect of a mass radio campaign on child mortality using facility utilisation data and the Lives Saved Tool (LiST): findings from a cluster randomised trial in Burkina Faso

**DOI:** 10.1136/bmjgh-2018-000808

**Published:** 2018-07-16

**Authors:** Joanna Murray, Roy Head, Sophie Sarrassat, Jennifer Hollowell, Pieter Remes, Matthew Lavoie, Josephine Borghi, Frida Kasteng, Nicolas Meda, Hermann Badolo, Moctar Ouedraogo, Robert Bambara, Simon Cousens

**Affiliations:** 1 Development Media International, London, UK; 2 Centre for Maternal Adolescent Reproductive and Child Health (MARCH), London School of Hygiene & Tropical Medicine, London, UK; 3 Department of Global Health and Development, Health Economics and Systems Analysis Group, London School of Hygiene & Tropical Medicine, London, UK; 4 Centre MURAZ, Bobo-Dioulasso, Burkina Faso; 5 Africsanté, Bobo Dioulasso, Burkina Faso; 6 Direction Générale des Études et des Statistiques Sectorielles (DGESS), Ministère de la Santé, Ouagadougou, Burkina Faso

**Keywords:** mass media, child mortality, cluster randomised controlled trial, Burkina Faso

## Abstract

**Background:**

A cluster randomised trial (CRT) in Burkina Faso was the first to demonstrate that a radio campaign increased health-seeking behaviours, specifically antenatal care attendance, health facility deliveries and primary care consultations for children under 5 years.

**Methods:**

Under-five consultation data by diagnosis was obtained from primary health facilities in trial clusters, from January 2011 to December 2014. Interrupted time-series analyses were conducted to assess the intervention effect by time period on under-five consultations for separate diagnosis categories that were targeted by the media campaign. The Lives Saved Tool was used to estimate the number of under-five lives saved and the per cent reduction in child mortality that might have resulted from increased health service utilisation. Scenarios were generated to estimate the effect of the intervention in the CRT study areas, as well as a national scale-up in Burkina Faso and future scale-up scenarios for national media campaigns in five African countries from 2018 to 2020.

**Results:**

Consultations for malaria symptoms increased by 56% in the first year (95% CI 30% to 88%; p<0.001) of the campaign, 37% in the second year (95% CI 12% to 69%; p=0.003) and 35% in the third year (95% CI 9% to 67%; p=0.006) relative to the increase in the control arm. Consultations for lower respiratory infections increased by 39% in the first year of the campaign (95% CI 22% to 58%; p<0.001), 25% in the second (95% CI 5% to 49%; p=0.010) and 11% in the third year (95% CI −20% to 54%; p=0.525). Diarrhoea consultations increased by 73% in the first year (95% CI 42% to 110%; p<0.001), 60% in the second (95% CI 12% to 129%; p=0.010) and 107% in the third year (95% CI 43% to 200%; p<0.001). Consultations for other diagnoses that were not targeted by the radio campaign did not differ between intervention and control arms. The estimated reduction in under-five mortality attributable to the radio intervention was 9.7% in the first year (uncertainty range: 5.1%–15.1%), 5.7% in the second year and 5.5% in the third year. The estimated number of under-five lives saved in the intervention zones during the trial was 2967 (range: 1110–5741). If scaled up nationally, the estimated reduction in under-five mortality would have been similar (9.2% in year 1, 5.6% in year 2 and 5.5% in year 3), equating to 14 888 under-five lives saved (range: 4832–30 432). The estimated number of lives that could be saved by implementing national media campaigns in other low-income settings ranged from 7205 in Burundi to 21 443 in Mozambique.

**Conclusion:**

Evidence from a CRT shows that a child health radio campaign increased under-five consultations at primary health centres for malaria, pneumonia and diarrhoea (the leading causes of postneonatal child mortality in Burkina Faso) and resulted in an estimated 7.1% average reduction in under-five mortality per year. These findings suggest important reductions in under-five mortality can be achieved by mass media alone, particularly when conducted at national scale.

**Key questions**

**What is already known?**

- Media campaigns can potentially reach a large audience at relatively low cost but have historically been poorly evaluated.
- A recent cluster randomised trial (CRT) in Burkina Faso found that a saturation-based radio campaign increased antenatal care attendance, health facility deliveries and primary care consultations for children under 5 years but was unable to detect a reduction in child mortality.

**What are the new findings?**

- Evidence from the CRT shows that the radio campaign substantially increased under-five consultations at primary health centres for malaria, pneumonia and diarrhoea, the leading causes of postneonatal child mortality in Burkina Faso.
- Using the Lives Saved Tool, the effect of the media campaign on healthcare seeking behaviours was estimated to reduce under-five deaths by an average of 7.1% per year.

**What do the new findings imply?**

- Substantial changes in health-seeking behaviours and reductions in under-five mortality can be achieved by saturation-based media campaigns, and these should belong in the mainstream of public health interventions.

## Introduction

Mass media campaigns have the potential to reach a large audience at a relatively low cost. As such, they have an important role to play in behaviour change communication to improve child survival in low-income and middle-income countries.^1^ However, there have been few attempts to rigorously measure the impact of mass media.^2^ A systematic review of evaluations of mass media interventions for child survival-related behaviours in low-income and middle-income countries reported that media campaigns can have a positive impact on a wide range of child health behaviours.^3^ The review highlighted scope for improvement in the often weak evaluations of media campaigns and lack of cost-effectiveness analyses to allow comparisons with other types of health intervention.^3^

Using the Lives Saved Tool (LiST), we previously modelled the potential impact of saturation-based media campaigns, estimating that they could reduce child mortality by 10%–20% at a cost per disability-adjusted life-year (DALY) that is as low as the most cost-effective child health interventions.^2 4^ We tested these predictions by conducting the first cluster randomised trial (CRT) to evaluate the impact that a radio campaign alone could have on child mortality (ClinicalTrial.gov Identifier: NCT01517230). As described elsewhere,^5^ the study clusters consisted of 14 rural geographical areas with high radio-listenership, each centred on a community radio station, with areas around towns excluded to remove populations with potential access to electricity and hence television. Seven clusters were randomly allocated to receive the intervention or control using pair-matched randomisation based on geographical proximity and radio listenership. A baseline survey conducted after randomisation revealed some important differences in the socioeconomic characteristics of the intervention and control clusters, particularly with regard to ethnicity, religion, distance to the nearest health facility and distance to the capital city (a marker of general level of development). Postneonatal under-five child mortality risk also differed between the intervention and control zones (see online supplementary appendix 1 for map and baseline characteristics of clusters). A cluster-level summary confounder score was calculated using principal component analysis (described elsewhere^6^) and used to control for imbalance between the groups.

10.1136/bmjgh-2018-000808.supp1 Supplementary file 1

The intervention consisted of a 35-month intensive radio campaign, running from March 2012 to January 2015, across seven community FM radio stations, which has been described in detail previously.^5^ Briefly, caregivers of children less than 5 years old were the primary target audience of the campaign, which covered a wide range of maternal and child health behaviours. Sixty-second radio spots were broadcast approximately 10 times per day, and 2-hour interactive (long-format) programmes were broadcast 5 days per week.

The endline trial results are presented elsewhere.^6^ Routine health facility data showed the intervention led to an increase in primary care consultations among children under-five, in all 3 years of the campaign (35% in year 1: p<0.001, 20% in year 2: p=0.003% and 16% in year 3: p=0.049, see online supplementary appendix 1a). The intervention also led to increases in antenatal care (ANC) attendances (6% in year 1: p=0.004% and 9% in year 2: p=0.026) and health facility deliveries (7% in year 1: p=0.004, 6% in year 2: P=0.003 and 9% in year 3: p<0.001). This constitutes the most rigorous evidence to date that a radio campaign alone can increase health facility utilisation for maternal and child health in a low-income setting.^6^

The study did not detect an impact of the campaign on the primary outcome of postneonatal child mortality. The power of the study was constrained by the limited number of clusters available. The original power calculations estimated the evaluation had 80% power to detect a 20% reduction in postneonatal child mortality and 54% power to detect a 15% reduction. However, rapidly declining child mortality in both arms and substantial between-cluster heterogeneity at baseline further reduced the power of the study to detect an effect on mortality.^6^ In this paper, we present further trial data on under-five consultations disaggregated by diagnosis. We then estimate the impact of the increased health service utilisation observed in the trial on under-five mortality in Burkina Faso using the LiST.

## Methods

### Routine health facility data

For 78 primary health facilities located across trial clusters (41 in control clusters), routine health facility data were obtained from the Direction Générale des Etudes et des Statistiques Sanitaires of the Ministry of Health in Burkina Faso.^6^ Monthly counts of all-cause under-five consultations were obtained from January 2011 to February 2016, and monthly counts of clinical diagnoses as reported by healthcare workers were obtained from January 2011 to December 2014. Reported diagnoses are based on clinical symptoms assessed by health workers and are not typically supported by diagnostic or laboratory test confirmation. Diagnoses were divided into the following categories of illnesses that were addressed by the radio campaign and correspond to the three leading causes of postneonatal under-five child mortality: malaria (complicated and uncomplicated), lower respiratory infections (pneumonia and bronchopneumonia) and diarrhoea. The remaining diagnoses were categorised as follows: upper respiratory tract infections (URTIs), malnutrition and ‘other’, included all diagnoses not captured by any diagnostic category listed above.

Health worker diagnoses were recorded, but the primary reason for presentation was not. Each child attending a health centre may have had multiple diagnoses recorded per consultation, so the number of diagnoses recorded does not equal the number of children presenting (online supplementary appendix 2b). To facilitate modelling of deaths averted using LiST, we therefore compressed the absolute numbers in each diagnosis category proportionally (online supplementary appendix 2c) to create compressed counts, summing to 100% of all-cause under-five consultations (online supplementary appendix 3)(figures 2–7).

### Analysis of routine data

Interrupted time-series analyses were conducted using mixed effects Poisson regression of compressed and uncompressed monthly counts of attendances per cluster, from January 2011 to December 2014, to assess the intervention effect by time period on under-five consultations for separate diagnosis categories. The model included fixed effects allowing for a long-term secular trend, for month of the year to account for seasonal variation, for intervention status of the cluster to account for systematic differences between arms at baseline, for confounder score and for intervention effect by period, with cluster treated as a random effect.^6^ To obtain 95% CIs and p values, we used bootstrap resampling (using the BC_a_ method and 1000 bootstrap replications).^7^

### The LiST modelling

Mortality impact was estimated using LiST.^8 9^ LiST allows users to model counterfactual scenarios in order to calculate the impact of a projected scale up of a health intervention on mortality outcomes and to compare the impacts of alternative scenarios. More detailed information about key inputs used by the tool, including sources of data on intervention effectiveness and cause-specific mortality by country, are provided in a series of journal supplements.^8 10–13^

### LiST analysis

**Figure 1:**
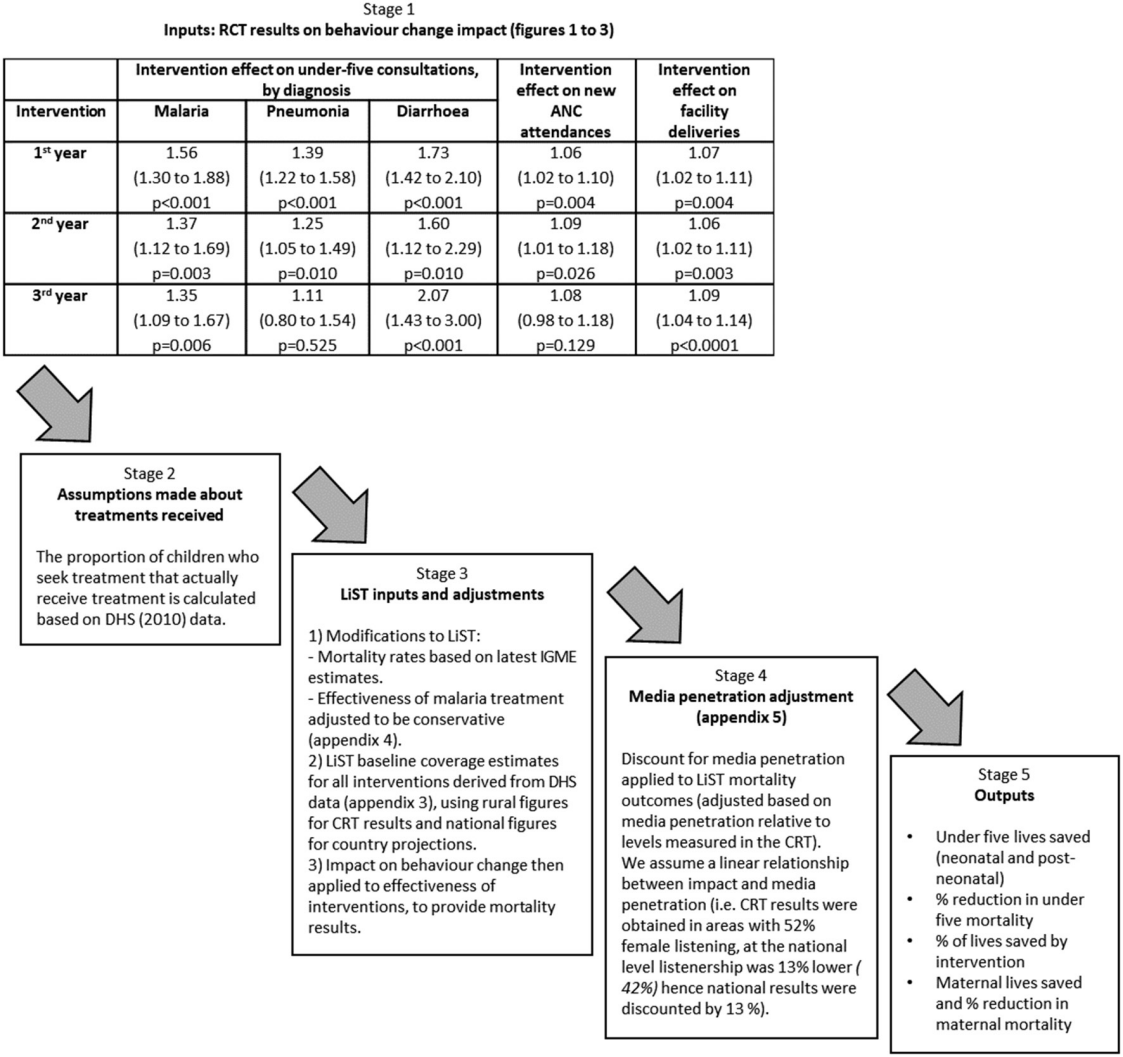
Stages of LiST modelling approach. ANC, antenatal care; CRT, cluster randomised trial; LiST, Lives Saved Tool; RCT, randomised controlled trial; DHS, Demographic Health Survey; IGME, United Nations Inter-Agency Group for Child Mortality.

We used health facility data from the CRT as the basis for modelling the effect of the radio intervention on child mortality (online supplementary appendix 2a). The baseline and endline coverage estimates used in this modelling can be found in online supplementary appendix 4 and are described in more detail below. A flow diagram summarising each stage of the described modelling approach and assumptions made is provided in figure 1.

Spectrum software V.5.63 was used for all analyses. The LiST package was used to model the mortality change in the intervention arm from 2011 (baseline) to 2014. We examined the effect of increasing health facility utilisation in line with the estimates from the CRT while holding the coverage of all other interventions constant at their 2011 level according to the base LiST projection data file for Burkina Faso (which uses DHS 2010 as its primary data source). For ANC, we applied increases of 6% in year 1, 9% in year 2 and 8% in year 3 to coverage of the three routine pregnancy interventions in LiST: tetanus toxoid vaccination, intermittent preventive treatment of malaria in pregnancy and syphilis detection and treatment. Coverage of all other antenatal interventions such as prevention of mother-to-child transmission of HIV, nutritional supplements or case management of pregnancy related disorders was not altered. For facility deliveries/skilled birth attendance, we modelled a 7% increase in year 1, 6% in year 2 and 9% in year 3 (online supplementary appendix 2a). Where the LiST programme projects any other lives saved due to any other interventions (such as measles vaccination or HIV treatment with time-varying coverage), these were subtracted from the lives saved totals as no media campaigning was conducted or impact measured on these interventions.

**Figure 2:**
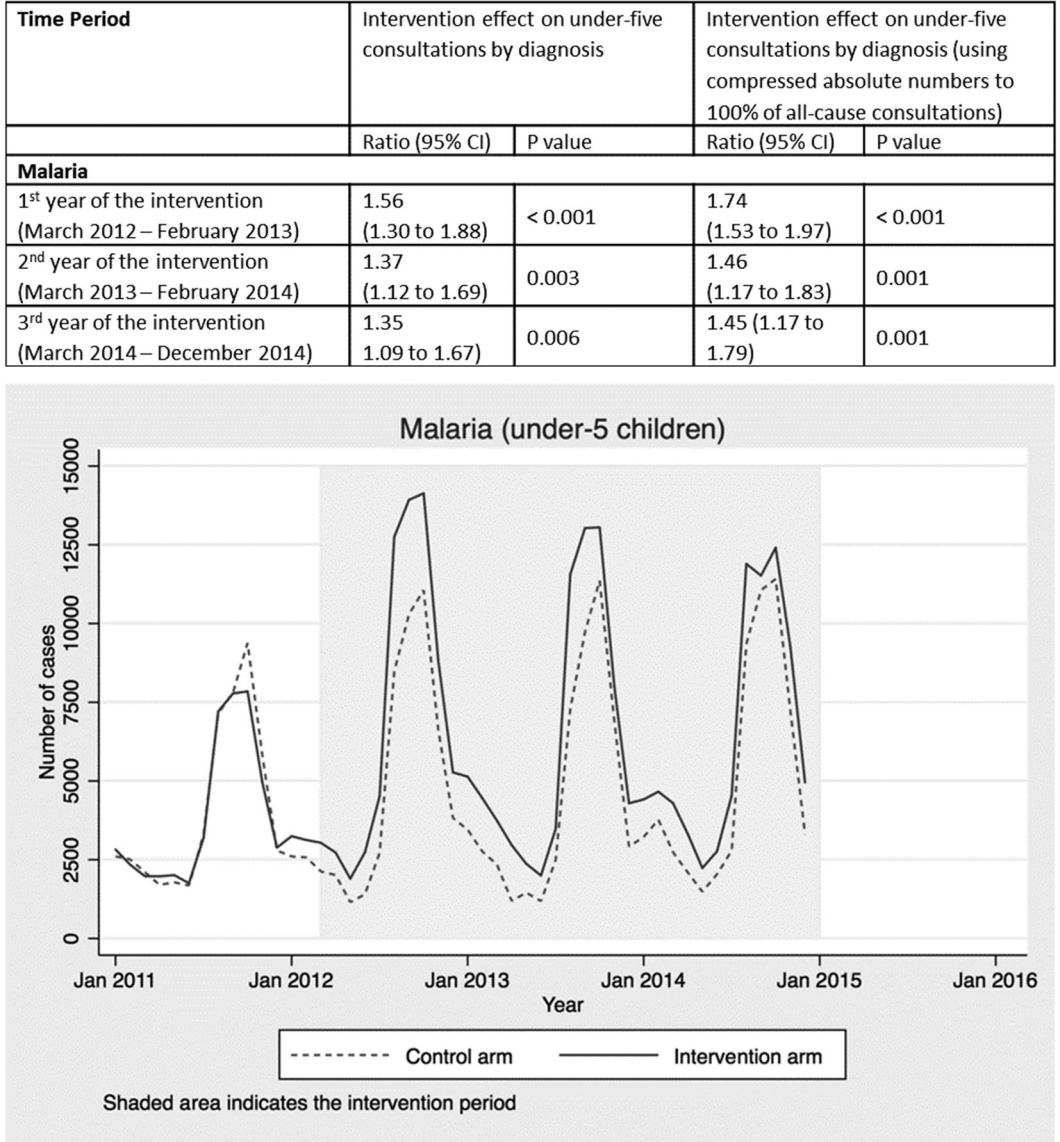
Intervention effect and absolute number of under-five consultations for malaria by time period and arm.

**Figure 3:**
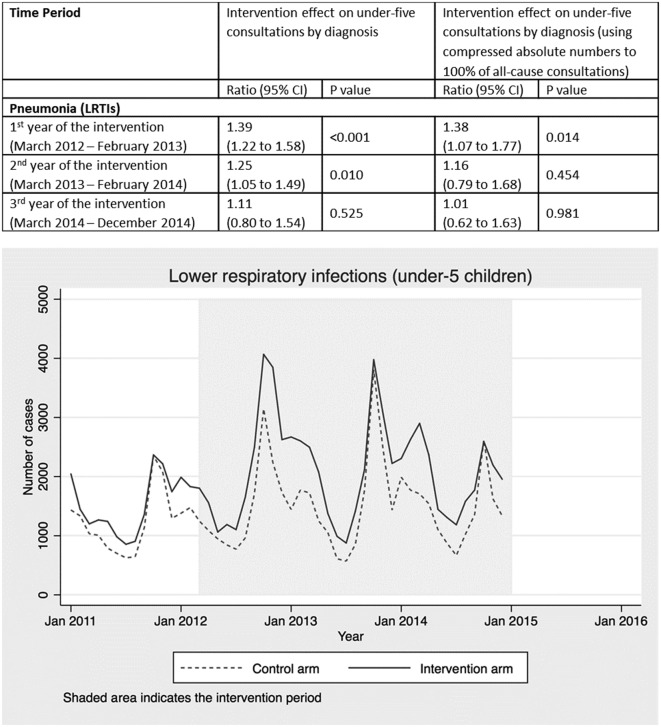
Intervention effect and absolute number of under-five consultations for pneumonia by time period and arm. LRTI, Lower respiratory tract infection.

**Figure 4:**
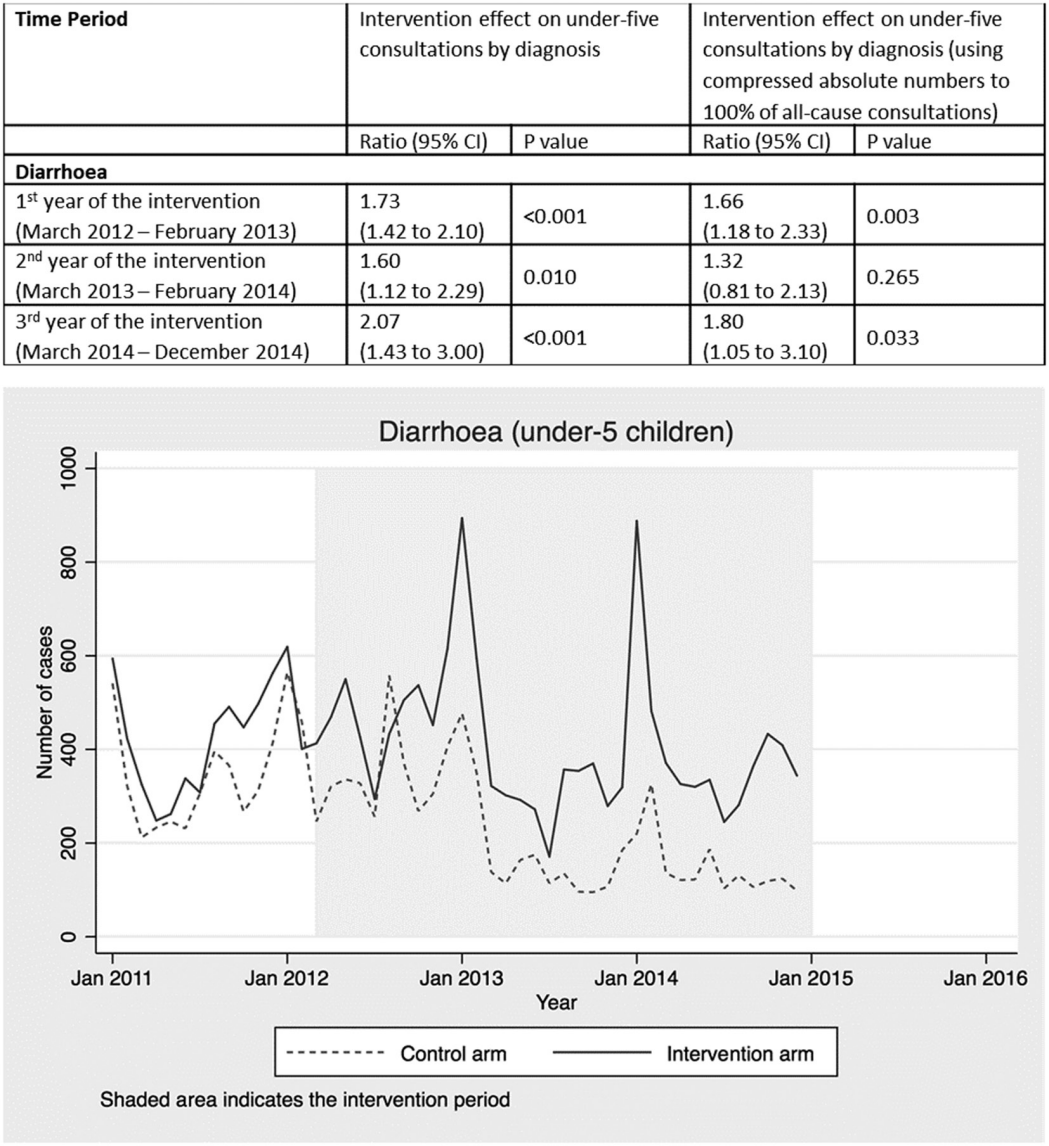
Intervention effect and absolute number of under-five consultations for diarrhoea by time period and arm.

Annual percentage increases in seeking treatment (figures 2–4) for each of the three main childhood illnesses targeted by the radio campaign, malaria, diarrhoea and pneumonia (the three primary causes of postneonatal under-five mortality in Burkina Faso), were applied to treatment coverage estimates in LiST.^14^ To do this, assumptions were made about the proportion of the increased number of children seeking treatment who *actually* received appropriate treatments. Data from the Demographic Health Survey (DHS) 2010 survey were used to estimate the proportion of children taken to health facilities (public primary health centres only) with symptoms of these illnesses who received effective treatments: artemisinin combination therapy (ACT) for malaria and oral rehydration solution (ORS) for diarrhoea. Where available, DHS data specifically relating to rural areas were used for the CRT scenario, since the campaign and evaluation were focused on rural areas only. The proportion of children taken to public primary health facilities who received effective treatments (54.3% of those with fever received an antimalarial and 34.5% of those with diarrhoea received ORS, according data for rural areas from DHS 2010) was then multiplied by the increase in facility attendances attributed to the radio intervention for each diagnosis group in each year (figures 2–4). This was then applied to the baseline LiST coverage values for the proportion of all children with malaria or diarrhoea who received treatment, adjusted for rural/national differences, also based on DHS 2010. For the oral antibiotics for pneumonia field, the LiST definition uses care-seeking for pneumonia at a health centre as a proxy for treatment with oral antibiotics. We therefore simply applied the increase in facility attendances for pneumonia attributed to the radio intervention in each year.^15^

To account for declining rates of under-five mortality in both arms throughout the trial period, we input separate under-five mortality rates for each year (2012–2014), based on the endline mortality survey (137 under-five deaths per 1000 live births in 2012, 126.5 in 2013 and 105.1 in 2014 in the intervention arm).^6^ We then obtained the corresponding total number of under-five deaths (occurring in the absence of the intervention) for each year, which served as the denominator to calculate the percentage reductions in mortality that occurred as a result of the lives saved by the intervention. For the national scale-up scenario, child mortality rates of 103.3 (2012), 97.7 (2013) and 92.8 (2014) per 1000 live births were assumed, based on the most recent (2017) Inter-Agency Group for Child Mortality (IGME) estimates.^16^ The percentage reduction in mortality was then calculated by dividing the estimated number of under-five lives saved by the total number of under five deaths (in the absence of the intervention) for each separate year. For all years, the distribution of under-five deaths by cause was based on the WHO 2011 estimates for Burkina Faso.^17^

LiST uses an estimated effectiveness of ACT treatment for malaria of 99%.^18^ Although ACT is the recommended treatment distributed to government primary care facilities in Burkina Faso, we assumed some children might not have received the correct, effective antimalarial and so we modelled a more conservative effectiveness of malaria treatments of 87%. This value was derived based on estimates of treatments received from the Malaria Indicators Survey 2014, consultation with the Burkina Faso Malaria Control Programme and estimated resistance rates of alternative malaria treatments that may have been prescribed (see online supplementary appendix 5).^19^ The modelled effectiveness for antibiotic treatment for pneumonia was 70% and for ORS was 93% (the LiST default values). For all three treatments modelled, the affected fraction (per cent of deaths due to a specific cause that are potentially able to be impacted by a specific intervention) for children aged 0–59 months was set at 1.0. All other data regarding demographics, population size, incidence of diseases, intervention coverage and effectiveness remained as provided in the LiST base projection data file for Burkina Faso, mostly using DHS 2010 data.

The impact of changes in intervention coverage on under-five and maternal mortality were modelled, and these national projections were then adjusted to reflect the population reached in the CRT, estimated using government population data to be 2.4 million (around 15% of the total population of Burkina Faso).^20^ We made a further adjustment to reflect radio penetration at national scale. To do this, we assumed that the number of people impacted would be directly proportional to the number of people exposed. We therefore adjust the mortality outcomes generated by the LiST modelling, using the figure for female radio listening in our intervention zones (52% in the endline survey) as a linear index. According to the 2010 DHS, the national figure for women across Burkina Faso listening to radio in the last week was 45.2%. We therefore applied a 13.1% reduction to the national mortality outcomes (reflecting the percentage reduction in female radio listenership from 52% to 45.2%).

### Sensitivity analysis

There is currently no single, standardised approach for calculating uncertainty bounds around LiST mortality estimates due to the challenge of determining the extent to which errors and biases in the different model inputs overlap and are correlated.^21^ We therefore conducted an additional sensitivity analysis by running projections using the upper and lower bounds of the 95% CI around the increases in health service utilisation reported in the CRT (online supplementary appendix 2). This produced the range of possible mortality reductions reported.

In order to be conservative in our scale-up modelling, we also applied a reduction to the estimated number of lives saved to reflect the reduction in impact that is likely to occur due to adaptation of the intervention for delivery at the national level. Qualitative implementation research captured during the campaign suggested spots were more strongly linked to changes in behaviour. The national scale-up is therefore a spots-only campaign at the same intensity as the spot broadcasts during the trial. In the absence of any data on which to base the estimated reduction in impact due to removal of long format programmes, we modelled a range, applying a 0%, 10% and 20% reduction to under-five lives saved.

### Future scale-up scenarios

Burkina Faso has a unique configuration of community radio stations with very low penetration of national media making it feasible to conduct a CRT, and such a study would be impossible to replicate in most countries. Hence, we explored generalisability of our findings by using LiST to explore whether the changes in healthcare-seeking behaviour demonstrated in Burkina Faso would have a similar impact on child mortality in other countries with different underlying patterns of treatment seeking and mortality. We followed a similar approach to model the impact of national media campaigns in five potential scale-up countries: Burkina Faso, Burundi, Malawi, Mozambique and Niger. These projections implicitly assume that the media campaign would be culturally adapted in each of the countries using the processes and principles used to develop the campaign in the CRT.^5^ These countries were selected because they vary in terms of their media landscape, population size, and rates and causes of child mortality. Health impacts in each country were derived from the care-seeking effects observed in the Burkina Faso trial and applied to LiST baseline coverage estimates for ANC, facility deliveries and treatment for childhood illnesses, as described above, for each separate country. For Burkina Faso, baseline coverage estimates were again based on the most recent DHS 2010 figures, as for projections described earlier. LiST was then used to project mortality effects for 3-year media campaigns in each country from 2018 to 2020. We made the assumption that the number of people impacted was directly proportional to the number exposed and therefore adjusted for media penetration levels, using DHS estimates of radio and/or television penetration in each country (see online supplementary appendix 6) and using the figure for female radio listening in Burkina Faso (52% as measured by the CRT endline survey)^6^ as a linear index (online supplementary appendix 6). Again, to estimate the reduction in impact due to removal of long-format programmes, we applied a 0%, 10% and 20% reduction to under-five lives saved.

## Results

### Routine health facility data

Consultations with a diagnosis of malaria increased significantly throughout the radio campaign (figure 2) by 56% in the first year (95% CI 30% to 88%; p<0.001), 37% in the second year (95% CI 12% to 69%; p=0.003) and 35% in the third year (95% CI 9% to 67%; p=0.006). Malaria was the most common diagnosis recorded for 55% of all under-five consultations across the 3-year period (online supplementary appendix 1c and 2).

Consultations with a diagnosis of lower respiratory infection increased by 39% in the first year of the campaign (95% CI 22% to 58%; p<0.001) 25% in the second year (95% CI 5% to 9%; p=0.010) and 11% in the third year (95% CI −20% to 54%; p=0.525) (figure 3). A pneumonia diagnosis was recorded for 18% of all under-five consultations across the 3-year period (online supplementary appendix 1c and 2).

Consultations with a diagnosis of diarrhoea increased by 73% in the first year (95% CI 42% to 110%; p<0.001), 60% in the second year (95% CI 12% to 129%; p=0.010) and 107% in the third year (95% CI 43% to 200%; p<0.001) (figure 4). A diarrhoea diagnosis was recorded for 3% of all under-five consultations across the 3-year period (online supplementary appendix 1c and 2).

**Figure 5:**
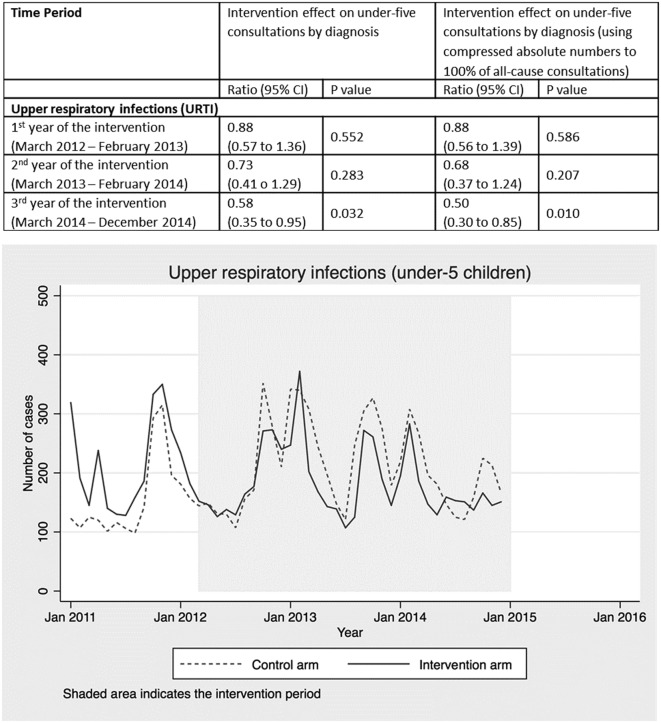
Intervention effect and absolute number of under-five consultations for URTI by time period and arm. URTI, upper respiratory tract infection.

**Figure 6:**
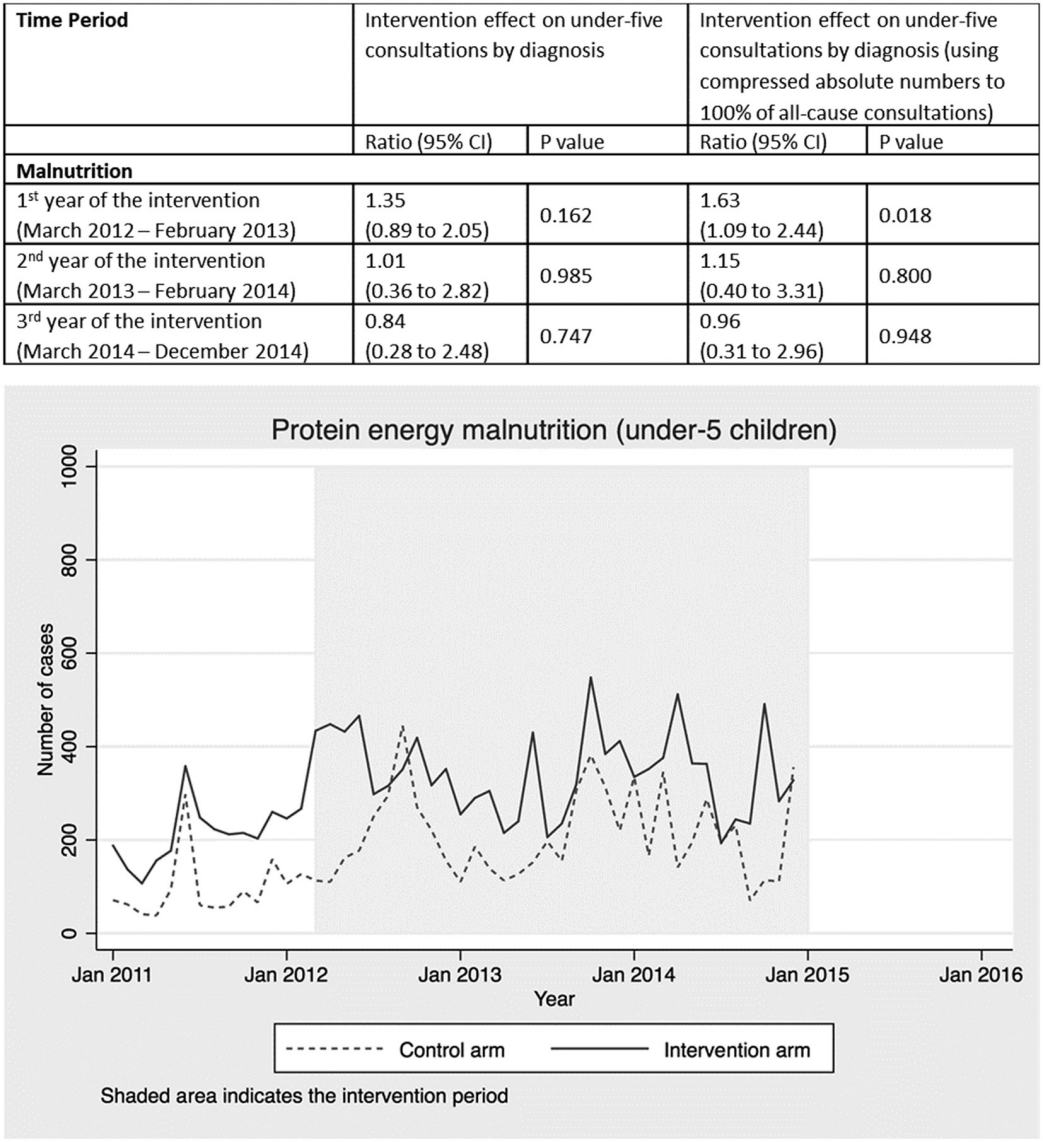
Intervention effect and absolute number of under-five consultations for malnutrition by time period and arm.

**Figure 7:**
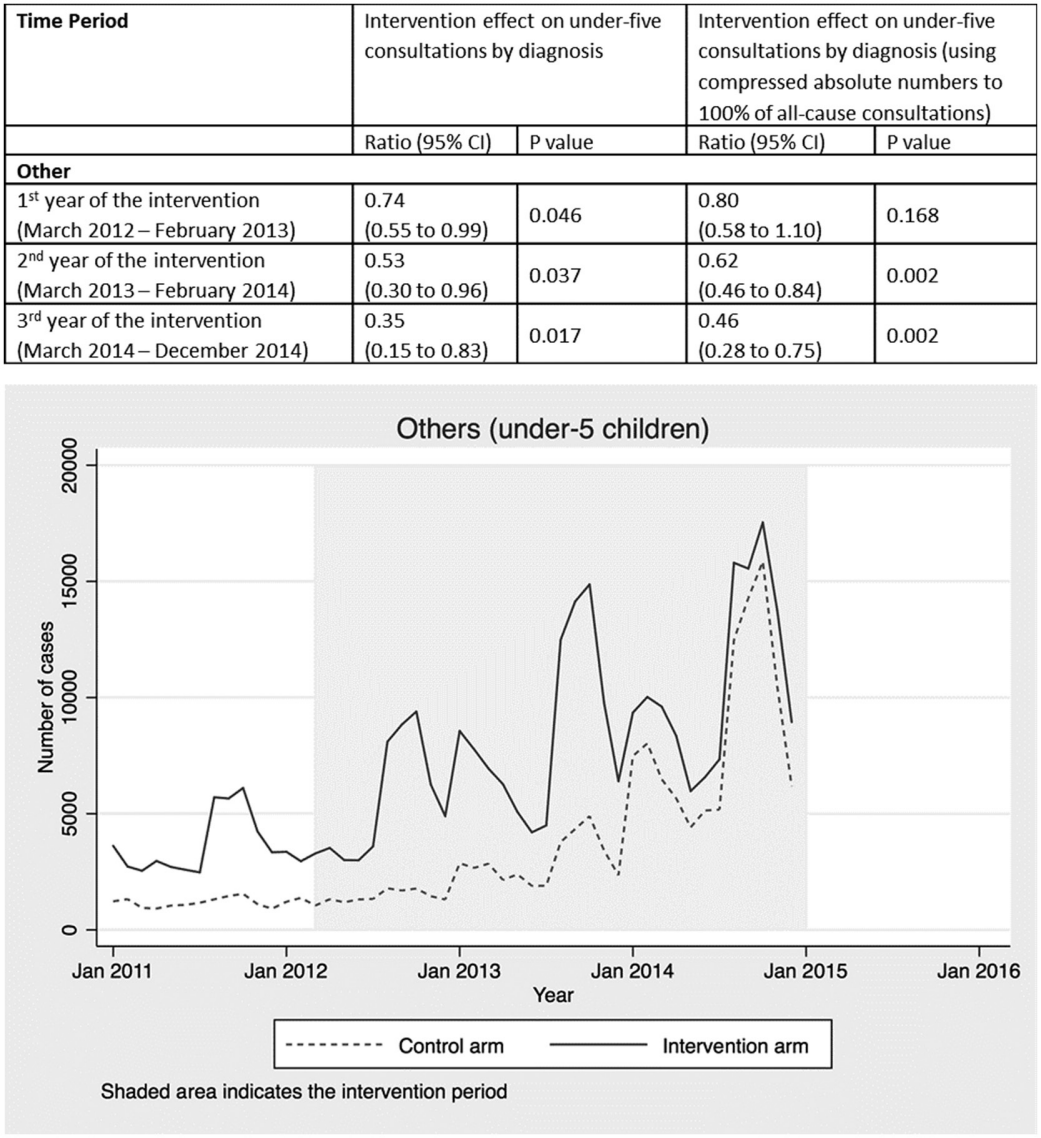
Intervention effect and absolute number of under-five consultations for other diagnoses by time period and arm.

Other diagnoses that were not targeted by the radio campaign, such as URTI (figure 5), malnutrition (figure 6) and other diagnoses (figure 7), showed no intervention effect. In the case of the other category, consultations rose in both arms but significantly more in the control arm.

### Lives saved modelling

The total number of under-five lives estimated to have been saved in areas covered by the radio campaign was 2967, with lower and upper bounds of 1110–5741 (table 1). From this modelling, we estimate the radio intervention reduced child mortality in the areas it covered by 9.7% (5.1%–15.1%) in the first year, 5.7% (0.2%–13.1%) in the second year and 5.5% (−0.1%–13.1%) in the third year.

**Table 1:** 2012–2014 projected lives saved by radio campaign in seven CRT intervention zones

Burkina Faso	2012	2013	2014	Total 2012–2014
Under-five lives saved*	1491 (777 to 2307)	817 (95 to 1880)	658 (239 to 1554)	2967 (1110–5741)
Percentage reduction in under- five mortality	9.7	5.7	5.5	7.1

*Estimated lower and upper bounds shown in brackets; totals may not agree with individual counts due to rounding.

Appropriate treatment of malaria accounted for the greatest reduction in mortality (61% of the lives saved) followed by antibiotics for treatment of pneumonia (18%) and delivery in a health centre/presence of a skilled birth attendant (16%). ORS for treatment of diarrhoea and ANC accounted for 5% and 1% of lives saved, respectively.

Extrapolating these results to the national level, adjusted by 13.1% to allow for lower radio-listenership, we estimate that the total number of under-five lives that would have been saved if the intervention had been delivered at a national scale was 14 888 over 3 years (with lower and upper bounds of 4832–30 432). This represents an estimated reduction in under-five mortality of 9.2% in year 1, 5.6% in year 2 and 5.5% in year 3 (an average reduction of 6.8% per year). After applying a further discount to account for adaptation of the intervention for the nationwide scale-up, the estimated number of lives that would have been saved by a national radio campaign is 13 400 (4349 to 27 389) when a 10% discount is applied, and 11 910 (3865 to 24 345) when a 20% discount is applied (table 2).

**Table 2:** 2012–2014 projected lives that could have been saved by a national radio campaign in Burkina Faso

Burkina Faso	2012	2013	2014
Under-five lives saved*	6690 (3413 to 10 288)	4143 (336 to 9413)	4055 (1083 to 10 731)
Percentage reduction in mortality	9.2	5.6	5.5
Under-five lives saved (10% discounting)	6021 (3072 to 9259)	3729 (302 to 8472)	3650 (975 to 9658)
Percentage reduction in mortality	8.3	5.1	5.0
Under-five lives saved (20% discounting)	5352 (2730 to 8230)	3314 (269 to 7530)	3244 (866 to 8585)
Percentage reduction in mortality	7.4	4.5	4.4

*Estimated lower and upper bounds shown in brackets.

Although the aim of DMI’s campaign was to reduce child deaths, our LiST analyses also suggest that a nationwide campaign could have saved maternal lives through increased uptake of antenatal and delivery care services. The estimated number of maternal lives saved in the intervention zones was 39 (range: 12–65) and if scaled up nationally would have been 227 (range: 72–376), representing an average 3% reduction in maternal mortality. The estimated number of under five lives that could be saved by implementing national media campaigns in other low income settings, ranged from 7205 in Burundi to 21 443 in Mozambique (table 3).

**Table 3:** Estimated number of under-five lives saved per year by a national media intervention in five low-income countries

2018–2020 LiST projections*	Year	Burkina Faso	Burundi	Malawi	Mozambique	Niger
Under-five lives saved	2018	4714 (7.7)	3171 (9.8)	7384 (20.7)	8519 (10.9)	6031 (6.7)
	2019	2851 (4.6)	1847 (5.5)	5664 (15.5)	4777 (6.0)	3142 (3.4)
	2020	2469 (3.9)	2187 (6.4)	6649 (17.9)	8147 (10.0)	4391 (4.6)
Under-five lives saved (with 10% discount)	2018	4242 (6.9)	2854 (8.8)	6645 (18.6)	7667 (9.8)	5428 (6.0)
	2019	2566 (4.1)	1662 (5.0)	5097 (14.0)	4299 (5.4)	2828 (3.0)
	2020	2223 (3.5)	1969 (5.8)	5984 (16.1)	7333 (9.0)	3952 (4.1)
Under-five lives saved (with 20% discount)	2018	3771 (6.1)	2537 (7.8)	5907 (16.5)	6815 (8.7)	4825 (5.4)
	2019	2281 (3.7)	1478 (4.4)	4531 (12.4)	3822 (4.8)	2513 (2.7)
	2020	1976 (3.1)	1750 (5.1)	5319 (14.3)	6518 (8.0)	3513 (3.7)

*Percentage reductions in mortality are in brackets.

## Discussion

Routine health facility data collected as part of a CRT in Burkina Faso provides strong evidence that a radio campaign alone significantly increased consultations at primary health facilities among under-fives with symptoms of malaria, pneumonia and diarrhoea (the three leading causes of postneonatal child mortality in Burkina Faso) as well as ANC and facility deliveries. Further supporting this evidence of an intervention effect, the data showed no difference between the intervention and control arms in consultations for diagnoses that were not targeted by the media campaign, such as upper respiratory infections, malnutrition and other childhood diagnoses. Using LiST, we estimate that increased health service utilisation attributed to the radio campaign should have resulted in 2967 under-five and 39 maternal lives saved. When scaled up to a national level, we estimate delivery of the intervention would have resulted in between 11 910 and 14 888 under-five and 182 to 227 maternal lives saved. These results suggest that mass media represent an important tool for changing health behaviours and improving maternal and child survival at scale, in low-income countries, dependent on radio listenership levels and service availability.

LiST has been widely used to model the impact of RMNCH interventions on health outcomes,^13 22–26^ including other programmes in Burkina Faso.^21 27 28^ Using a well validated tool to model the effect of the intervention on mortality enables us to estimate the potential mortality impact of our campaign. The estimated reduction in under-five mortality (including the lower and upper bounds) corresponds to an effect size that the mortality survey in the CRT was not powered to detect (and which any future studies would struggle to detect) and is compatible with the CIs around the estimated mortality reduction in the trial (risk ratio 1.0, 95% CI 0.82 to 1.22).^6^ Furthermore, LiST enables the estimation of maternal lives saved which the CRT could never have been powered to measure.

This modelling further represents an important contribution to the evidence base for social and behaviour change communication, since it has facilitated a health economic analysis.^3^ An accompanying study has used these findings to assess the cost-effectiveness of mass media campaigns, analysing both the costs of the radio intervention implemented for the CRT as well as the costs of delivering it at a national scale in Burkina Faso and other countries). Mass media is an unusual intervention in that it is usually easier to deliver at scale than within the limitations of a cluster trial. Mass media campaigns could potentially be used in any similar setting with minimal demand generation initiative and good service availability, perhaps supplementing existing successful integrated community case management activities. Kasteng *et al* have estimated that the incremental provider cost-effectiveness of a national media campaign in Burkina Faso during the trial period would have been $15 per DALY averted, with a societal cost (including the additional costs to households of changed care-seeking) of $38 per DALY averted.^20^ For national campaigns in five African countries from 2018 to 2020, the projected provider cost ranged from $7 to $27 per DALY averted.

Our modelling was based on results from a CRT, using routine health facility data that was well powered to detect changes in care seeking and was collected as part of the most rigorous evaluation of a mass media intervention to have been conducted in a developing country.^6^ There is extensive evidence, derived from non-randomised studies from multiple low-income countries, that mass media campaigns with adequate exposure can change health-related behaviours affecting child survival.^3^ Our projections for other countries illustrate that the impact seen in Burkina Faso is potentially generalisable to other countries with different patterns of treatment seeking and mortality. However, these projections assume that the intervention (with cultural adaptation) would be equally effective at increasing healthcare seeking in other countries, which may not be the case.

Health facilities in Burkina Faso adopted the District Health Information System (DHIS) 2 data management system in 2013. In 2014, data submissions from health facilities were over 95% complete,^29^ but there is limited information available on the quality of the data collected. A further limitation is that the system does not provide precise denominator data.^6^

The average number of diagnoses per consultation increased in both arms over time, from 1.23 to 2.17 in the control arm and from 1.51 to 2.29 in the intervention arm (online supplementary appendix 2b). For this reason, it was necessary to compress the absolute numbers of diagnoses in each month to 100% of the total number of consultations to facilitate modelling. By far the largest contributor to this pattern was the other diagnosis category (not targeted by the campaign), which rose more rapidly in the control arm (online supplementary appendix 2c). The reasons for this are unclear.

In our modelling, we made adjustments to reflect the fact that not all sick children taken to a health facility will necessarily receive treatment (perhaps because a health worker did not adhere to guidance, or treatment was not actually required or due to supply-side constraints). We estimated this based on 2010 DHS data (or LiST automatically estimates this in the case of pneumonia) on the proportions of children with fever or diarrhoea taken to a health facility who received an ACT or ORS, respectively. We used the DHS since it is the largest, most representative population-based data available on which to base these figures and, as such, is also the basis for most intervention coverage figures used in LiST. However, by relying on DHS data from 2010 to estimate treatment coverage, we may be underestimating the proportion of children who received treatment for malaria and diarrhoea during the study period. Our modelling may overestimate the proportion of children who receive antibiotics for pneumonia, as LiST uses care-seeking for pneumonia at a health centre as a proxy for the proportion receiving oral antibiotic treatment for pneumonia.

Given the rapid declines in child mortality reported in the CRT mortality survey, it is likely that since 2010 investments in the health system in Burkina Faso have improved the availability of treatments. Indeed, the WHO 2014 Service and Availability Readiness Assessment (SARA) report suggests good availability of key life-saving treatments in primary health facilities: 91% for malaria treatment, 82% for ORS sachets and 83% for antibiotics (amoxicillin), based on their survey of 659 primary health facilities across Burkina Faso.^30^ This more recent survey of health centres provides a reliable assessment of treatment availability but does not provide an estimate of the proportion of symptomatic children who receive the available treatments. We were therefore reliant on the DHS to estimate this and such population-based surveys are not without their limitations. Retrospective recall of illness episodes and reporting of received treatments by parents using a 2-week recall period may be prone to errors and even these large-scale surveys are subject to the challenges of small samples for many childhood illnesses. No other national data on treatments received for all major causes of childhood illness during the study period were available. However, an ongoing evaluation related to the use of Integrated Management of Childhood Illness guidelines in one region of Burkina Faso found that only 3 out of 819 cases (0.4%) of uncomplicated malaria diagnosed by health workers did not receive an ACT (S Sarrassat, personal communication). This was based on observations of health workers during consultations in primary care facilities rather than recall of parents.

Several assumptions are inherent in our modelling approach, and we have limited data available to test their validity in the context of Burkina Faso, including regarding the causes of child deaths and the effectiveness of different treatments prescribed. We also made an assumption that we did not increase the proportion of children attending health centres who did not actually require treatment. We do know the clinical diagnoses ascribed by health workers to the increased consultations, and we know that these reflected the three leading causes of postneonatal child mortality in Burkina Faso (malaria, pneumonia and diarrhoea). It is possible that some people travelled to the health centre for trivial reasons, but we would expect these are recorded and captured within the ‘other’ diagnosis category rather than malaria, pneumonia or diarrhoea diagnoses. We also know that parents living in intervention areas tended to have much further to travel to their closest health facility than parents living in the control areas (a median of 6.3 km compared with 2.5 km in the control areas) usually by foot or bicycle. Moreover, we can only hypothesise that it is unlikely parents would make the journey to health centres unless their child was quite unwell.

## Conclusion

Evidence from a CRT shows that a child health radio campaign resulted in increased under-five consultations at primary health facilities for malaria, pneumonia and diarrhoea (the leading causes of postneonatal child mortality in Burkina Faso). Our modelling suggests that increased health service utilisation attributed to the radio campaign, resulted in important reductions in child and maternal mortality of 7.1% and 3%, respectively, with an estimated 2967 under-five and 39 maternal lives saved, in a low-income rural setting in Burkina Faso.

## References

[1] Fox E, Obregón R Population-level behavior change to enhance child survival and development in low- and middle-income countries. J Health Commun 2014;19 Suppl 1:3–9. 10.1080/10810730.2014.934937 25207445 PMC4205918

[2] Head R, Murray J, Sarrassat S, et al Can mass media interventions reduce child mortality? Lancet 2015;386:97–100. 10.1016/S0140-6736(14)61649-4 25684587

[3] Naugle DA, Hornik RC Systematic review of the effectiveness of mass media interventions for child survival in low- and middle-income countries. J Health Commun 2014;19(suppl 1):190–215. 10.1080/10810730.2014.918217 PMC4205927 25207453

[4] Laxminarayan R, Mills AJ, Breman JG, et al Advancement of global health: key messages from the Disease Control Priorities Project. Lancet 2006;367:1193–208. 10.1016/S0140-6736(06)68440-7 16616562

[5] Murray J, Remes P, Ilboudo R, et al The Saturation+ Approach to Behavior Change: Case Study of a Child Survival Radio Campaign in Burkina Faso. Glob Health Sci Pract 2015;3:544–56. 10.9745/GHSP-D-15-00049 26681703 PMC4682581

[6] Sarrassat S, Meda N, Badolo H, et al Effect of a mass radio campaign on family behaviours and child survival in Burkina Faso: a repeated cross-sectional, cluster-randomised trial. Lancet Glob Health 2018;6:e330–41. 10.1016/S2214-109X(18)30004-4 29433668 PMC5817351

[7] Efron B, Tibshirani RJ An introduction to the bootstrap. New York: Chapman and Hall, 1993:184.

[8] Walker N, Tam Y, Friberg IK Overview of the Lives Saved Tool (LiST). BMC Public Health 2013;13(Supp 3):S1 10.1186/1471-2458-13-S3-S1 PMC3847271 24564438

[9] The lives saved tool. http://livessavedtool.org/

[10] Walker N, Fischer-Walker C, Bryce J, et al Standards for CHERG reviews of intervention effects on child survival. Int J Epidemiol 2010;39(S1):i21–31. 10.1093/ije/dyq036 20348122 PMC2845875

[11] Fox MJ, Martorell R, van den Broek N, et al Assumptions and methods in the Lives Saved Tool (LiST). Introduction. BMC Public Health 2011;11(Suppl 3):I1 10.1186/1471-2458-11-S3-I1 PMC3231881 21501425

[12] Walker N The Lives Saved Tool in 2013: new capabilities and applications. BMC Public Health 2013;11(Supp 3):S1. 10.1186/1471-2458-13-S3-S1 PMC3847271 24564438

[13] Walker N, Yenokyan G, Friberg IK, et al Patterns in coverage of maternal, newborn, and child health interventions: projections of neonatal and under-5 mortality to 2035. Lancet 2013;382:1029–38. 10.1016/S0140-6736(13)61748-1 24054534

[14] Liu L, Johnson HL, Cousens S, et al Global, regional, and national causes of child mortality: an updated systematic analysis for 2010 with time trends since 2000. Lancet 2012;379:2151–61. 10.1016/S0140-6736(12)60560-1 22579125

[15] The Lives Saved Tool Manual and Help File. http://www.livessavedtool.org/images/documents/manuals/LiST-Help-English-March-2017.pdf.

[16] IGME. Child Mortality Estimates(. 2017 http://www.childmortality.org/files_v21/download/IGME%20report%202017%20child%20mortality%20final.pdf (accessed Jan 2018).

[17] WHO Global Health Observatory data repository. http://apps.who.int/gho/data/view.main.ghe3002015-BFA?lang=en (accessed Jan 2018). 10.1080/02763869.2019.1693231 32069199

[18] Thwing J, Eisele TP, Steketee RW Protective efficacy of malaria case management and intermittent preventive treatment for preventing malaria mortality in children: a systematic review for the Lives Saved Tool. BMC Public Health 2011;11(Suppl 3):S14 10.1186/1471-2458-11-S3-S14 PMC3231887 21501431

[19] Malaria Indicators Survey (MIS). Burkina Faso, 2014.

[20] Kasteng F, Murray J, Cousens S, et al Cost-effectiveness and economies of scale of a mass radio campaign to promote household life-saving practices in Burkina Faso. BMJ Global Health 2018 doi: 10.1136/bmjgh-2018-000809 [Epub ahead of print 2018]. 10.1136/bmjgh-2018-000809 PMC6058168 30057798

[21] Munos M, Guiella G, Roberton T, et al Independent Evaluation of the Rapid Scale-Up Program to Reduce Under-Five Mortality in Burkina Faso. Am J Trop Med Hyg 2016;94:584–95. 10.4269/ajtmh.15-0585 26787147 PMC4775895

[22] Akseer N, Salehi AS, Hossain SM, et al Achieving maternal and child health gains in Afghanistan: a Countdown to 2015 country case study. Lancet Glob Health 2016;4:e395–413. 10.1016/S2214-109X(16)30002-X 27198844

[23] Kanyuka M, Ndawala J, Mleme T, et al Malawi and Millennium Development Goal 4: a Countdown to 2015 country case study. Lancet Glob Health 2016;4:e201–14. 10.1016/S2214-109X(15)00294-6 26805586

[24] Afnan-Holmes H, Magoma M, John T, et al Tanzania’s countdown to 2015: an analysis of two decades of progress and gaps for reproductive, maternal, newborn, and child health, to inform priorities for post-2015. Lancet Glob Health 2015;3:e396–409. 10.1016/S2214-109X(15)00059-5 26087986

[25] Bhutta ZA, Das JK, Bahl R, et al Can available interventions end preventable deaths in mothers, newborn babies, and stillbirths, and at what cost? Lancet 2014;384:347–70. 10.1016/S0140-6736(14)60792-3 24853604

[26] Darmstadt GL, Bhutta ZA, Cousens S, et al Evidence-based, cost-effective interventions: how many newborn babies can we save? Lancet 2005;365:977–88. 10.1016/S0140-6736(05)71088-6 15767001

[27] Marsh A, Munos M, Baya B, et al Using LiST to model potential reduction in under-five mortality in Burkina Faso. BMC Public Health 2013;13 Suppl 3(Suppl 3):S26 10.1186/1471-2458-13-S3-S26 24564341 PMC3847624

[28] Johri M, Ridde V, Heinmüller R, et al Estimation of maternal and child mortality one year after user-fee elimination: an impact evaluation and modelling study in Burkina Faso. Bull World Health Organ 2014;92:706–15. 10.2471/BLT.13.130609 25378724 PMC4208477

[29] Ministere de la Santé de Burkina Faso. Annuaire statistique 2014: Direction générale des études et des statistiques sectorielles (DGESS), 2015.

[30] WHO Service and Availability Readiness Assessment (SARA) for Burkina Faso, 2014.

