# Supplementary material for: Modelling the effect of a mass radio campaign on child mortality using facility utilisation data and the Lives Saved Tool (LiST): findings from a cluster randomised trial in Burkina Faso
Source: BMJ Glob Health. 2018 Jul 16;3(4):e000808. doi: 10.1136/bmjgh-2018-000808 (PMC6058176; doi:10.1136/bmjgh-2018-000808)
Supplement: Supplementary file 1 [file bmjgh-2018-000808supp001.docx]

# Supplementary file

List of appendices

[Appendix 1: Map and characteristics of intervention and control zones 2](#_Toc512439668)

[Appendix 2a: Intervention effect on attendances at primary care facilities by year (routine data) 4](#_Toc512439669)

[Appendix 2b: Mean number of diagnoses per consultation by arm and time period 4](#_Toc512439670)

[Appendix 2c: Absolute numbers of under-five consultations by diagnosis, time period and arm. 5](#_Toc512439671)

[Appendix 3: All-cause under-five consultations 6](#_Toc512439672)

[Appendix 4: Baseline and endline intervention coverage figures modelled in LiST. 8](#_Toc512439673)

[Appendix 5: Estimated effectiveness of malaria treatment 10](#_Toc512439674)

[Appendix 6: Adjustments for media penetration in each scale-up country scenario. 10](#_Toc512439675)

## A**ppendix 1: Map and characteristics of intervention and control zones**

**Figure 1: Map of intervention and control clusters showing pair matched randomisation based on geography and radio penetration rates**


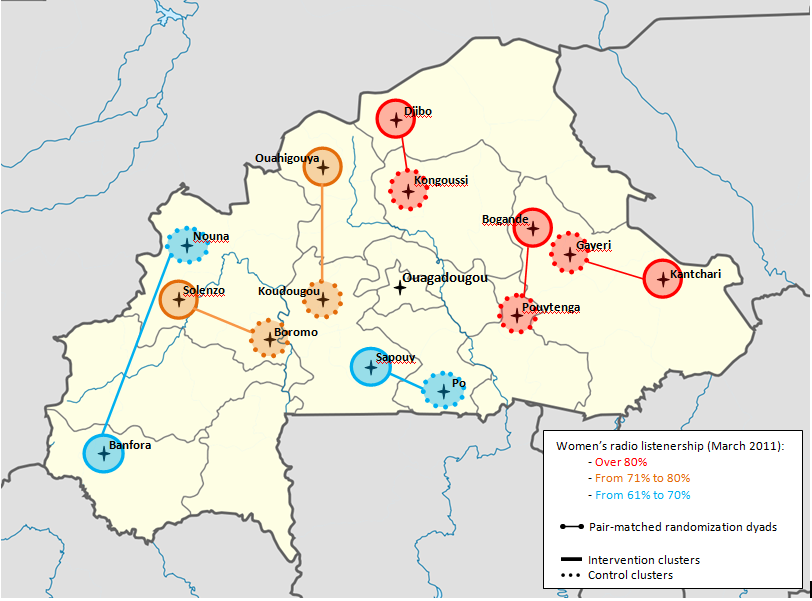


**Mothers' socio-demographic characteristics at baseline**

|  | | | **Control** | **Intervention** |
| --- | --- | --- | --- | --- |
|  | | | (N = 2,567) | (N = 2,476) |
| Age in years (mean) | | | 28.9 | 28.4 |
| 3 years or more residence duration in the village (%) | | | 91.3 | 93.9 |
| Ethnicities (%)  Mossi | | | 42.1 | 30.1 |
| Gourmantche | | | 11.5 | 26.9 |
| Gourounssi | | | 22.1 | 3.2 |
| Peulh | | | 6.5 | 17.0 |
| Gouin/Karaboro/Turka | | | 0.2 | 13.9 |
| Marka/Dafing/Dioula | | | 8.4 | 3.5 |
| Bwaba/Bobo | | | 7.5 | 3.3 |
| Other | | | 1.6 | 2.1 |
| Religion (%)  Muslim | | | 47.2 | 60.1 |
| Catholic/Protestant | | | 45.0 | 26.4 |
| Animist | | | 7.8 | 13.5 |
| School attendance (%) | | | 15.6 | 10.2 |
| Household socio-economic status (%)  1 (poorest) | | | 14.2 | 18.8 |
| 2 | | | 16.7 | 20.4 |
| 3 | | | 19.3 | 20.3 |
| 4 | | | 21.7 | 20.1 |
| 5 (least poor) | | | 28.1 | 20.3 |
| Radio ownership (%)  No radio | | | 20.5 | 13.2 |
| Radio in the compound | | | 16.8 | 22.4 |
| Radio in the household | | | 62.8 | 64.5 |
| Married (%) | | | 97.1 | 98.3 |
| Polygamous union (%) | | | 39.6 | 40.3 |
| Two or more under-five children (%) | | | 39.4 | 46.4 |
| Age of the youngest child in months (mean) | | | 21.1 | 19.4 |
| Distance to the nearest health facility (%)  < 2 km | | | 39.5 | 18.3 |
| 2-5 km | | | 33.2 | 28.2 |
| > 5 km | | | 27.4 | 53.4 |
|  |  |  |  |  |
|  |  |  |  |  |

## Appendix 2a: Intervention effect on attendances at primary care facilities by year (routine data)


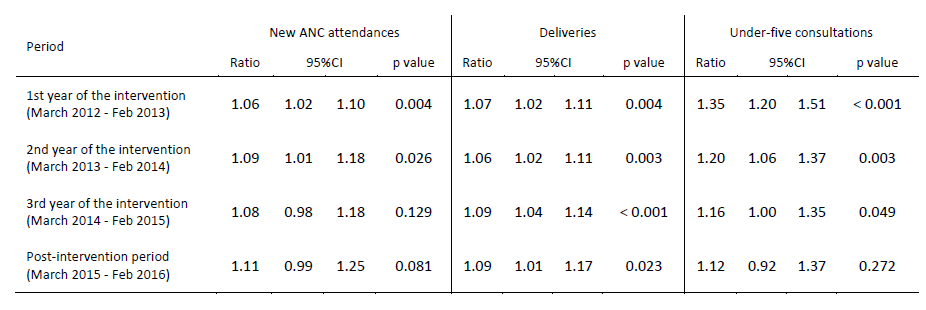


## Appendix 2b: Mean number of diagnoses per consultation by arm and time period

|  | Control arm | | | Intervention arm | | |
| --- | --- | --- | --- | --- | --- | --- |
| Time period | Under-5 consultations | All diagnoses | Mean number of diagnoses/ consultation | Under-5 consultations | All diagnoses | Mean number of diagnoses/ consultation |
| Pre-intervention period (Mar 2011 - Feb 2012) | 68,681 | 84,418 | **1.23** | 79,852 | 120,662 | **1.51** |
| 1st year of the intervention (March 2012 - Feb 2013) | 83,022 | 102,762 | **1.24** | 111,758 | 189,270 | **1.69** |
| 2nd year of the intervention (March 2013 - Feb 2014) | 82,559 | 125,994 | **1.53** | 103,191 | 213,755 | **2.07** |
| 3rd year of the intervention (March 2014 - Dec 2014) | 73,028 | 158,625 | **2.17** | 89,363 | 204,260 | **2.29** |

## Appendix 2c: Absolute numbers of under-five consultations by diagnosis, time period and arm.

| **Time Period** | **Control arm** | | **Intervention arm** | | ***Ratio of ratios*** |
| --- | --- | --- | --- | --- | --- |
|  | Absolute number | Ratio to baseline | Absolute number | Ratio to baseline |  |
| **Malaria** | | | | | |
| Pre-intervention period  (March 2011 – February 2012) | 48,700 | **1.00** | 47,970 | **1.00** | **-** |
| 1^st^ year of the intervention  (March 2012 – February 2013) | 55,902 | **1.15** | 79,389 | **1.65** | **1.44** |
| 2^nd^ year of the intervention  (March 2013 – February 2014) | 53,866 | **1.11** | 73,561 | **1.53** | **1.39** |
| 3^rd^ year of the intervention  (March 2014 – December 2014) | 53,523 | **1.10** | 67,197 | **1.40** | **1.27** |
| **Pneumonia (LRTIs)** | | | | | |
| Pre-intervention period  (March 2011 – February 2012) | 14,523 | **1.00** | 17,959 | **1.00** | **-** |
| 1^st^ year of the intervention  (March 2012 – February 2013) | 17,910 | **1.23** | 26,681 | **1.49** | **1.20** |
| 2^nd^ year of the intervention  (March 2013 – February 2014) | 19,318 | **1.33** | 25,560 | **1.42** | **1.07** |
| 3^rd^ year of the intervention  (March 2014 – December 2014) | 13,854 | **0.95** | 19,302 | **1.07** | **1.13** |
| **Diarrhoea** | | | | | |
| Pre-intervention period  (March 2011 – February 2012) | 4,006 | **1.00** | 4,954 | **1.00** | **-** |
| 1^st^ year of the intervention  (March 2012 – February 2013) | 4,222 | **1.05** | 6,182 | **1.25** | **1.18** |
| 2^nd^ year of the intervention  (March 2013 – February 2014) | 1,869 | **0.47** | 4,408 | **0.89** | **1.91** |
| 3^rd^ year of the intervention  (March 2014 – December 2014) | 1,247 | **0.31** | 3,428 | **0.69** | **2.22** |
| **URTI** | | | | | |
| Pre-intervention period  (March 2011 – February 2012) | 1,950 | **1.00** | 2,497 | **1.00** | **-** |
| 1^st^ year of the intervention  (March 2012 – February 2013) | 2,511 | **1.29** | 2,435 | **0.98** | **0.76** |
| 2^nd^ year of the intervention  (March 2013 – February 2014) | 2,878 | **1.48** | 2,229 | **0.89** | **0.60** |
| 3^rd^ year of the intervention  (March 2014 – December 2014) | 1,800 | **0.92** | 1,524 | **0.61** | **0.66** |
| **Malnutrition** | | | | | |
| Pre-intervention period  (March 2011 – February 2012) | 1,191 | **1.00** | 2,672 | **1.00** | **-** |
| 1^st^ year of the intervention  (March 2012 – February 2013) | 2,495 | **2.09** | 4,378 | **1.64** | **0.78** |
| 2^nd^ year of the intervention  (March 2013 – February 2014) | 2,605 | **2.19** | 3,981 | **1.49** | **0.68** |
| 3^rd^ year of the intervention  (March 2014 – December 2014) | 2,048 | **1.72** | 3,389 | **1.27** | **0.74** |
| **Other** | | | | | |
| Pre-intervention period  (March 2011 – February 2012) | 14,048 | **1.00** | 44,610 | **1.00** | **-** |
| 1^st^ year of the intervention  (March 2012 – February 2013) | 19,722 | **1.40** | 70,205 | **1.57** | **1.12** |
| 2^nd^ year of the intervention  (March 2013 – February 2014) | 45,458 | **3.24** | 104,016 | **2.33** | **0.72** |
| 3^rd^ year of the intervention  (March 2014 – December 2014) | 86,153 | **6.13** | 109,420 | **2.45** | **0.40** |
|  |  |  |  |  |  |

## Appendix 3: All-cause under-five consultations

For all-cause under-5 consultations data were obtained from January 2011 up to February 2016.^[[1]](#footnote-1)^ For diagnoses, data were obtained from January 2011 up to December 2014.

As a consequence, the analyses of all-cause under-five consultations and consultations by diagnosis are not directly comparable.

- In analysis A below (ratio to baseline of the absolute number of diagnoses by arm), comparison to baseline (March 2011 - February 2012) of the third year of the intervention (March 2014 – December 2014) is not directly comparable with the comparison to baseline presented in appendix 1a (for which there is complete data for the third year of the intervention (March 2014 – February 2015). Comparison to baseline in the post-intervention period (March 2015-February 2016) cannot be reported by diagnosis.
- For analysis B (interrupted time series analysis) with respect to all-cause under-5 consultations, this difference in time period for which data were obtained results in some changes to the estimated intervention effects because the model includes both monthly effects and a term for secular trends, and the different set of data affects the estimates of both of these components of the model and hence the estimated intervention effects.

**Analysis A: Absolute numbers of all-cause under-5 consultations by time period and by arm**

|  | Control arm | | Intervention arm | | Ratio of ratios |
| --- | --- | --- | --- | --- | --- |
| Time period | Absolute number | Ratio to baseline | Absolute number | Ratio to baseline |  |
| Pre-intervention period (Mar 2011 - Feb 2012) | 68,681 | **1.00** | 79,852 | **1.00** | **-** |
| 1st year of the intervention (March 2012 - Feb 2013) | 83,022 | **1.21** | 111,758 | **1.40** | **1.16** |
| 2nd year of the intervention (March 2013 - Feb 2014) | 82,559 | **1.20** | 103,191 | **1.29** | **1.08** |
| 3rd year of the intervention (March 2014 - Dec 2014) | 73,028 | **1.06** | 89,363 | **1.12** | **1.05** |

**Analysis B: Intervention effect by time period on all-cause under-5 consultations (time series analysis)**

| Time period | Ratio | 95%CI | | p |
| --- | --- | --- | --- | --- |
| 1st year of the intervention (March 2012 - Feb 2013) | **1.32** | 1.18 | 1.47 | < 0.001 |
| 2nd year of the intervention (March 2013 - Feb 2014) | **1.15** | 1.01 | 1.31 | 0.042 |
| 3rd year of the intervention (March 2014 - Dec 2014) | **1.10** | 0.95 | 1.27 | 0.202 |

**Under- five consultations (any diagnosis) by time period and arm**

]

## Appendix 4: Baseline and endline intervention coverage figures modelled in LiST.

***For CRT scenario (rural DHS data****)*

| **Intervention Coverage (%)** | **2011** | **2012** | **2013** | **2014** |
| --- | --- | --- | --- | --- |
| Tetanus toxoid vaccination | 88.0 | 93.3 | 95.9 | 95.0 |
| IPTp | 38.5 | 40.8 | 42.0 | 41.6 |
| Syphilis detection & treatment | 21.3 | 22.6 | 23.2 | 23.0 |
| Health facility delivery | 60.8 | 65.1 | 64.4 | 66.3 |
| Skilled birth attendant | 60.8 | 65.1 | 64.4 | 66.3 |
| ORS for treatment of diarrhoea | 18.9 | 23.2 | 21.0 | 24.1 |
| Oral antibiotics for treatment of pneumonia | 52.7 | 72.7 | 61.1 | 53.2 |
| ACTs for treatment of malaria | 32.6 | 45.7 | 40.7 | 40.6 |
|  |  |  |  |  |

**Lower Bound**

| **Intervention Coverage (%)** | **2011** | **2012** | **2013** | **2014** |
| --- | --- | --- | --- | --- |
| Tetanus toxoid vaccination | 88.0 | 89.8 | 88.9 | 86.2 |
| IPTp | 38.5 | 39.3 | 38.9 | 37.7 |
| Syphilis detection & treatment | 21.3 | 21.7 | 21.5 | 20.9 |
| Health facility delivery | 60.8 | 62.0 | 62.0 | 63.2 |
| Skilled birth attendant | 60.8 | 62.0 | 62.0 | 63.2 |
| ORS for treatment of diarrhoea | 18.9 | 20.1 | 18.3 | 19.2 |
| Oral antibiotics for treatment of pneumonia | 52.7 | 56.4 | 41.6 | 32.7 |
| ACTs for treatment of malaria | 32.6 | 42.0 | 35.6 | 35.6 |
|  |  |  |  |  |

**Upper Bound**

| **Intervention Coverage (%)** | **2011** | **2012** | **2013** | **2014** |
| --- | --- | --- | --- | --- |
| Tetanus toxoid vaccination | 88.0 | 96.8 | 100.0 | 100.0 |
| IPTp | 38.5 | 42.4 | 45.4 | 45.4 |
| Syphilis detection & treatment | 21.3 | 23.4 | 25.1 | 25.1 |
| Health facility delivery | 60.8 | 67.5 | 67.5 | 69.3 |
| Skilled birth attendant | 60.8 | 67.5 | 67.5 | 69.3 |
| ORS for treatment of diarrhoea | 18.9 | 27.6 | 26.3 | 32.6 |
| Oral antibiotics for treatment of pneumonia | 52.7 | 93.3 | 88.5 | 85.9 |
| ACTs for treatment of malaria | 32.6 | 49.8 | 47.3 | 46.6 |
|  |  |  |  |  |

***For the national scale-up scenario (national DHS data)***

| **Intervention Coverage (%)** | **2011** | **2012** | **2013** | **2014** |
| --- | --- | --- | --- | --- |
| Tetanus toxoid vaccination | 88.0 | 93.3 | 95.9 | 95.0 |
| IPTp | 38.5 | 40.8 | 42.0 | 41.6 |
| Syphilis detection & treatment | 21.3 | 22.6 | 23.2 | 23.0 |
| Health facility delivery | 66.3 | 70.9 | 70.3 | 72.3 |
| Skilled birth attendant | 66.3 | 70.9 | 70.3 | 72.3 |
| ORS for treatment of diarrhoea | 21.2 | 26.3 | 23.7 | 27.3 |
| Oral antibiotics for treatment of pneumonia | 56.0 | 77.3 | 65.0 | 56.6 |
| ACTs for treatment of malaria | 35.1 | 49.7 | 44.2 | 44.0 |
|  |  |  |  |  |

**Lower Bound**

| **Intervention Coverage (%)** | **2011** | **2012** | **2013** | **2014** |
| --- | --- | --- | --- | --- |
| Tetanus toxoid vaccination | 88.0 | 89.8 | 88.9 | 86.2 |
| IPTp | 38.5 | 39.3 | 38.9 | 37.7 |
| Syphilis detection & treatment | 21.3 | 21.7 | 21.5 | 20.9 |
| Health facility delivery | 66.3 | 67.6 | 67.6 | 69.0 |
| Skilled birth attendant | 66.3 | 67.6 | 67.6 | 69.0 |
| ORS for treatment of diarrhoea | 21.2 | 22.6 | 20.5 | 21.6 |
| Oral antibiotics for treatment of pneumonia | 56.0 | 59.9 | 44.2 | 34.7 |
| ACTs for treatment of malaria | 35.1 | 45.5 | 38.4 | 38.4 |
|  |  |  |  |  |

**Upper Bound**

| **Intervention Coverage (%)** | **2011** | **2012** | **2013** | **2014** |
| --- | --- | --- | --- | --- |
| Tetanus toxoid vaccination | 88.0 | 96.8 | 100.0 | 100.0 |
| IPTp | 38.5 | 42.4 | 45.4 | 45.4 |
| Syphilis detection & treatment | 21.3 | 23.4 | 25.1 | 25.1 |
| Health facility delivery | 66.3 | 73.6 | 73.6 | 75.6 |
| Skilled birth attendant | 66.3 | 73.6 | 73.6 | 75.6 |
| ORS for treatment of diarrhoea | 21.2 | 31.4 | 29.9 | 37.3 |
| Oral antibiotics for treatment of pneumonia | 56.0 | 99.1 | 94.1 | 91.3 |
| ACTs for treatment of malaria | 35.1 | 54.2 | 51.4 | 50.7 |
|  |  |  |  |  |

## Appendix 5: Estimated effectiveness of malaria treatment

According to the 2014 Malaria Indicators Survey (MIS) in Burkina Faso, **62.3%** of children (aged 0-59 months) with febrile illness taken to a health centre received treatments that were not ACT. Resistance to these treatments means they are likely to be less effective than ACTs, but in the absence of recent data from Burkina Faso from which to estimate this, we reduced the effectiveness for this proportion of febrile children receiving treatment to 80%. For the remaining 37.7% who were reported to have received ACT treatment for malaria, we assumed 99% effectiveness (as per the LiST default). This resulted in an overall modelled effectiveness for malaria treatment of 87%.

## Appendix 6: Adjustments for media penetration in each scale-up country scenario.

| **Scale-up Country** | **Media Penetration (among women)** | **Data Source** | **Adjustment applied to impact modelling*** |
| --- | --- | --- | --- |
| Burkina Faso | 45.2% weekly radio listening | DHS 2010 | -13% |
| Burundi | 57.9% weekly radio listening | DHS 2010 | +12% |
| Malawi | 57.3% weekly radio listening | DHS 2010 | +10% |
| Mozambique | 60.0% weekly radio listening† | DHS 2011 | +15% |
| Niger | 36.2% weekly radio listening | DHS 2012 | -30% |

* We adjusted the mortality outcomes generated by the LiST modelling using the figure for female radio listening in Burkina Faso (52% as measured by the CRT endline survey) as a linear index. For example, for Niger, the national radio-listenership was estimated to be 36.2% compared with 52% in the Burkina Faso CRT. The adjustment applied to the Mozambique projections was therefore -30%, calculated as (36.2% - 52%)/52%

† There are huge discrepancies between the two most recent estimates for female radio listening in national surveys conducted in Mozambique: 42.5% in the 2011 DHS, 78% in the 2009 AIS.  We have no reason to believe radio listening in Mozambique really did change that much between these two surveys and suspect this vast difference is a result of seasonal variation in the time the surveys were conducted.  We have therefore taken the midpoint of these two figures, ie (42.5+78%)/2=60%.

1. Sarrassat S, Meda N, Badolo H, et al, Evaluation of the effect of a mass radio campaign on family behaviours and child survival in Burkina Faso: Findings from a repeated cross sectional cluster randomised trial. *Lancet Global Health* 2018; **6**: e330–41 [↑](#footnote-ref-1)
